# Influence of Fluid Cell Design on the Frequency Response of AFM Microcantilevers in Liquid Media

**DOI:** 10.3390/s8095927

**Published:** 2008-09-25

**Authors:** Ramin Motamedi, Paula M. Wood-Adams

**Affiliations:** Center for Applied Research on Polymers and Composites (CREPEC), Department of Mechanical and Industrial Engineering, Concordia University, 1455 de Maisonneuve Blvd West, EV013.210, Montreal, Quebec, Canada H3G 1M8; E-Mail: r_motame@encs.concordia.ca

**Keywords:** AFM, microcantilever, frequency response, fluid cell, liquid

## Abstract

A study of the frequency response of AFM microcantilevers in liquid media contained in a commercial fluid cell is presented. Such systems exhibit complicated dynamics which are often not well described by available theories. Their dynamic behavior has a direct effect on the use of the AFM in dynamic mode while imaging in liquid or while extracting the rheological properties of the fluid. We explore the issues related to the design of the cantilever holder/fluid cell and propose an approach for evaluating, minimizing and recognizing the ultimate limitations of commercial cantilever holders. A technique for estimating the frequency response spectrum of the fluid cell itself from experimental data is presented. This spectrum can then be used to evaluate whether or not the fluid cell is suited for the desired purpose.

## 1. Introduction

Since the invention of the atomic force microscope (AFM) [1], many applications have been developed from imaging and scanning of biologic and non-biologic surfaces in vacuum [2], air, and liquid [3, 4] to more recently, measuring and determining the rheological properties of fluids surrounding the AFM cantilever [5, 6]. In many of these applications, the AFM is used in its dynamic mode, meaning that the cantilever is excited such that it oscillates at a frequency close to its primary natural frequency. The use of the AFM in the dynamic mode is challenging in liquid media because of the complex hydrodynamic force acting on the cantilever and therefore affecting its frequency response. Also several factors that originate from the design of the cantilever holder significantly affect the frequency response. Therefore, understanding the influence of each of these issues is necessary for the reliable operation of AFM in liquid media. Here we will explore the issues related to the design of the cantilever holder and propose an approach for evaluating, minimizing and recognizing the ultimate limitations of commercial cantilever holders.

There are three main techniques to excite an AFM cantilever: thermally, acoustically and magnetically. In a liquid environment the response of the cantilever strongly depends on the excitation technique. In the case thermal excitation [7, 8], the cantilever response or thermal noise is the result of random collisions from the Brownian motion of the surrounding fluid molecules. In this technique, the cantilever is excited directly and consequently a smooth vibration response, related only to the properties of the cantilever and the fluid, is observed. In the magnetic excitation technique [9], a microcantilever magnetized either by attaching a magnetic particle [10] or coating with a magnetic material [11] is excited by an external magnetic field. This is another direct excitation method providing a smooth vibration response. In comparison, the acoustic technique [4] is not a direct method. In this technique, the cantilever is excited through movement of its base by a piezoelectric actuator. The actuator is usually placed directly under the cantilever chip in the tip holder used in air or vacuum, while it is usually located away from the cantilever base in the fluid cell which is used for liquid media. As will be explained in detail later, the response of the cantilever to acoustic excitation, in a liquid environment, contains many spurious peaks which do not correspond to the natural frequencies of the cantilever and are rather related to the design of the fluid cell. It should also be noted that there are some other techniques [12, 13] for excitation of the AFM cantilever, which are not as common as the techniques discussed above.

Although the thermal and magnetic driving techniques produce smoother cantilever responses, they have some drawbacks which make working with acoustic excitation desirable. Firstly, these techniques require additional hardware such as a signal conditioner, a data acquisition system, special cantilevers, and a magnetic field system making these techniques more complex and costly. Secondly, in the magnetic technique, the fluid is heated by the electromagnetic field and the magnetic coating changes the vibrational properties and bending angle of the cantilever. For these reasons, many studies have been aimed at understanding and removing the redundant peaks in the response of the cantilever to acoustic excitation.

Putman *et al.* [4], who were the pioneers in introducing tapping mode atomic force microscopy in liquid media, were the first faced with these extra frequency peaks. They realized that any changes in the liquid cell system, such as changing its geometry, its material, the working liquid, and more importantly the amount of liquid, affect the positions and amplitudes of the resonances. Schaffer *et al.* [14] observed the same phenomena and based on their observations on the responses of different cantilevers in the same liquid environment, they proposed the hypothesis that “the cantilever response spectrum is the product of a fluid drive spectrum, which depends only on the cantilever module and fluid, and the thermal noise spectrum, which depends only on the cantilever and fluid”. Their hypothesis was supported by measuring the fluid drive spectra of three different cantilevers in the same environment and showing that their shapes are very similar. Moreover, they showed experimentally that the mode shapes of the vibrating cantilever are independent of the fluid drive spectrum and depend only on the vibrational characteristics of the cantilever in the fluid. Other researchers, who used different types of AFMs and fluid cells which in some cases were made in-house, also reported the appearance of spurious peaks [15-17]. This indicates that there are some common difficulties in the design of fluid cells.

Although the effects of the various design problems on the cantilever response were previously recognized, the exact relationships were not understood and improvement of the frequency response based on control of these factors has not previously been considered. Instead efforts were focused on other approaches. Tamayo *et al.* [18] mixed the standard driving signal with a feedback signal from the cantilever response such that they could increase the quality factor of the cantilever oscillations by up to three orders of magnitude. However their technique is very sensitive to viscosity variations and is limited by small temperature fluctuations. Rogers *et al.* [19] used another approach. They attached a piezoelectric microactuator over the axial surface of a microcantilever and insolated it from the conductive liquid medium using a fluoropolymer coating. In this way they could excite the microcantilever by applying a direct force, resulting in the disappearance of redundant peaks. However, like the magnetic coated cantilevers, the vibrational properties and bending angle of their cantilevers are changed.

Beside these practical investigations, a lot of effort has been focused on the evaluation of cantilever response theoretically. Schaffer *et al.* [14] proposed a simple model for the behavior of an oscillating cantilever in liquid media based on the assumption that the beam is driven by a uniform harmonic pressure, in phase with the spatial vibration, over its surface. Other researchers have developed theoretical models with more realistic assumptions. For example, Jai *et al.* [20] considered the cantilever as a point mass and spring in their modeling. They showed that for cantilevers having low quality factors, the displacement of the cantilever base is comparable to the cantilever oscillation amplitude. Therefore, in this case, the free end of the cantilever has a movement equal to the summation of the base displacement and the cantilever oscillation amplitude. Sader [8] proposed a general theoretical model with more rigorous assumptions. He considered the cantilever as a continuous mass system which can be excited by an arbitrary driving force. He simplified his model for the case of thermal noise which is well accepted and widely used. More recently, Xu and Raman [21] derived simple models based on transfer functions to describe the response of a cantilever to thermal, magnetic and ideal acoustic excitations (acoustic excitation is ideal when the base of the cantilever is moved in a controlled manner). They also studied experimentally the responses of the cantilever to these excitation techniques in liquid media using an Agilent AFM and fluid cell. They reached to the conclusion that in acoustic excitation the response of cantilever is the result of two mechanisms: a) structure-born excitation and b) fluid-born excitation. The structure-born excitation is due to the oscillation of the cantilever base while the fluid-born excitation is due to an unsteady fluid motion caused by the large moving surface of the cantilever base and fluid cell. The first mechanism is ideal acoustic excitation but when it is combined with the other mechanism, spurious peaks are observed in the cantilever response.

Also, because of the particular design of commercial fluid cells, it is impossible to apply ideal acoustic excitation to the cantilever which causes in an even more complicated frequency response. In this work, we apply some simple modifications to a widely used commercial fluid cell from Veeco [22] (MTFML model) in an effort to approximate ideal acoustic excitation and in this way investigate the frequency response of the cantilever in this cell without fluid born excitation and certain design related aspects. We will show that the vibration of the fluid cell body is the most significant disturbance in the observed frequency response.

## 2. Theory

**Figure 1.**
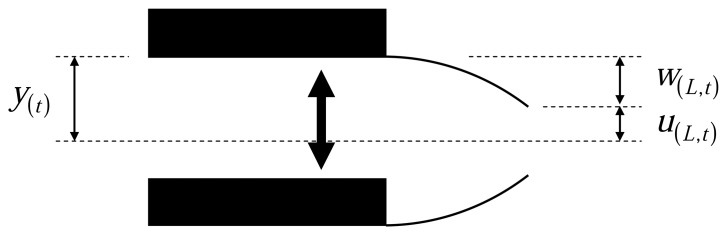
Cantilever movement in acoustic excitation. *u_(x_*_,_*_t)_* is the absolute tip motion and *w_(x_*_,_*_t)_* is the relative tip motion to the base motion *y_(t)_*.

In ideal acoustic excitation, the cantilever response is due to the movement of cantilever base, as shown schematically in Figure 1. Here, we follow the approaches of Sader [8] and Xu [21] for the hydrodynamic drag force and the theoretical response of the cantilever respectively.

Since the internal friction of the cantilever itself is negligible compared to fluid damping, the governing equation for the deflection of the cantilever can be written in the following form:

(1) $$EI\frac{\partial^{4}u\left(x,t\right)}{\partial x^{4}}+\rho_{c}A\frac{\partial^{2}u\left(x,t\right)}{\partial t^{2}}=f_{h}\left(u,\dot{u}\right)$$

where *u(x,t)* is the transverse cantilever deflection, *EI* is its flexural rigidity, *ρ_c_* is the mass density, *L* is the length, *A* =*b* × *h* is the area of the cross section, b and h are the width and thickness of the cantilever, respectively, and *f_h_* is the hydrodynamic resistance per unit length to cantilever motion. For this equation the boundary conditions are:

(2) $$\begin{array}{llll} u\left(0,t\right)=y\left(t\right), & \frac{\partial u\left(0,t\right)}{\partial x}=0, & \frac{\partial^{2}u\left(L,t\right)}{\partial x^{2}}=0, & \frac{\partial^{3}u\left(L,t\right)}{\partial x^{3}}=0 \end{array}$$

where *y(t)* is the base motion. We consider *w(x,t)* as the tip motion relative to the base, *w(x,t)*=*u(x,t)-y(t)*, and substitute for *u(x,t)* in the above equations. Then in the governing equation an additional term appears in the right side as an external force which is related to the inertial forces of the cantilever beam and the boundary condition at the base will change to *w(0,t)*=0. Now by applying the Fourier transform on the above equations we find in the frequency domain:

(3) $$EI\frac{\partial^{4}W\left(x,\omega \right)}{\partial x^{4}}-\rho_{c}\;A\omega^{2}W\left(x,\omega \right)=F_{h}\left(x,\omega \right)+\rho_{c}\;A\omega^{2}Y\left(\omega \right)$$

(4) $$\begin{array}{cccc} W\left(0,\omega \right)=0, & \frac{\partial W\left(0,\omega \right)}{\partial x}=0, & \frac{\partial^{2}W\left(L,\omega \right)}{\partial x^{2}}=0, & \frac{\partial^{3}W\left(L,\omega \right)}{\partial x^{3}}=0 \end{array}$$

To continue, we require a general form for the hydrodynamic force. Because the amplitude of vibration of the cantilever is very small, the hydrodynamic force on each point of the cantilever can be approximated by the hydrodynamic force that would be applied on an infinitely long rigid beam that oscillates transversely with the same amplitude, *w(x,t)*, in the fluid. This force was determined by Stokes [23] for a circular cylinder and Sader [8] modified it for rectangular cantilevers using an empirical correction function. The general form of this force is:

(5) $$F_{h}\left(x,\omega \right)=\frac{\pi }{4}\rho_{f}\;b^{2}\omega^{2}\Gamma \left(\omega \right)W\left(x,\omega \right)$$

where *ρ_f_* is the density of the surrounding fluid, *b* is the width of the cantilever and *Γ(ω)* is the hydrodynamic function that can be obtained from solving the momentum equations for the surrounding fluid.

The solution of equation 3 can be written in the form of:

(6) $$W(x,\omega )=\sum_{i=1}^{\infty }C_{i}\left(\omega \right)\psi_{i}\left(x\right)$$

where *C_i_(ω)* is the complex magnitude of the i^th^ mode, and *ψ_i_(x)* is the normalized eigenfunction of the i^th^ vibrational mode of the undamped cantilever. The eigenfunctions are normalized in a way that *ψ_i_(L)*=1 and have the orthogonality properties:

(7) $$\begin{array}{ll} \int_{0}^{1}\psi_{i}^{\prime \prime \prime \prime }\psi_{j}\;dx=\int_{0}^{l}\psi_{i}^{\prime \prime }\psi_{j}^{\prime \prime }\;dx=\int_{0}^{l}\psi_{i}\psi_{j}\;dx=0 & \;\left(i\neq j\right) \end{array}$$

After substituting equations 5 and 6 in equation 3, we can have the following equation by multiplication of the result by *ψ_i_(x)* and integrating over the length of the cantilever:

(8) $$\frac{C_{i}\left(\omega \right)}{Y\left(\omega \right)}=\frac{\omega^{2}\left(\rho_{c}A+\frac{\pi }{4}\;\rho_{f}\;b^{2}\Gamma_{\text{rect}}\right)\times \beta_{i}L}{EI{\left(\lambda_{i}/L\right)}^{4}\times \alpha_{i}L-\omega^{2}\left(\rho_{c}A+\frac{\pi }{4}\;\rho_{f}\;b^{2}\Gamma_{\text{rect}}\right)\times \alpha_{i}L}$$

where $\alpha_{i}L=\int_{0}^{L}\psi_{i}^{2}dx$, $\beta_{i}L=\int_{0}^{L}\psi_{i}dx$ and *λ_i_* is the i^th^ modal wavelength. herefore, given the amplitude of excitation, *Y(w)*, we can determine the deflection of the cantilever at each point. For simplicity, we can define the following transfer function using Equation 8 and 6:

(9) $$\begin{array}{lll} {\text{T}}_{\text{c}}\;\left(\text{x},\omega \right) & = & \frac{\text{W}\left(\text{x},\omega \right)}{\text{Y}\left(\omega \right)}\\ & = & \sum_{\text{i}=1}^{\infty }\left(\frac{\omega^{2}\left(\rho_{\text{c}}\text{A}+\frac{\pi }{4}\rho_{\text{f}}{\text{b}}^{2}\Gamma_{\text{rect}}\right){\times \beta }_{\text{i}}\text{L}}{\text{EI}{\left(\lambda_{\text{i}}/\text{L}\right)}^{4}{\times \alpha }_{\text{i}}\text{L}-\omega^{2}\left(\rho_{\text{c}}\text{A}+\frac{\pi }{4}\rho_{\text{f}}{\text{b}}^{2}\Gamma_{\text{rect}}\right){\times \alpha }_{\text{i}}\text{L}}\psi_{\text{i}}\left(\text{x}\right)\right) \end{array}$$

It should be noted that the quantity measured by AFM is in fact the inclination of the cantilever. For this case, the theoretical response is simply the spatial derivative of the cantilever deflection:

(10) $$\frac{\partial W\left(x,\omega \right)}{\partial x}=\sum_{i=1}^{\infty }C_{i}\left(\omega \right)\frac{\partial \psi_{i}\left(x\right)}{\partial x}$$

## 3. Experimental Studies

**Figure 2.**
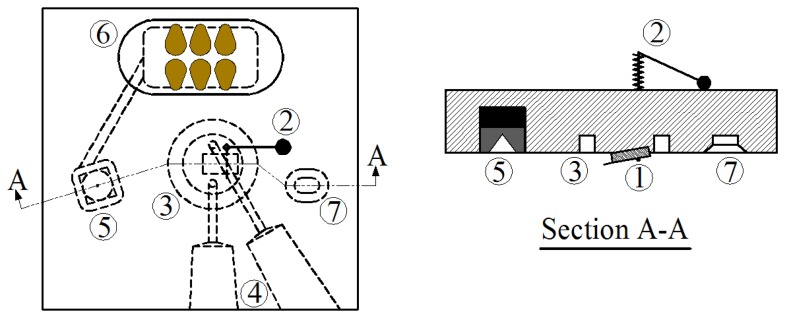
Schematic of a fluid cell. In this picture (1) is the cantilever, (2) is the clip and spring, (3) is the circular groove for o-ring, (4) are the inlet and outlet channels for exchanging liquids, (5) is the moving support, (6) is the connecting chip, and (7) is the fixed support.

Figure 2 is a schematic of the commercial fluid cell from Veeco (MTFML model) which can be used for tapping mode, force modulation, and contact mode experiments in liquids. The main purpose of using the fluid cell is to insulate and separate the piezoelectric actuator from conductive fluids. In this cell, the microcantilever chip (1) is placed in a small groove close to the middle of the bottom of the fluid cell and is fixed to the cell by a clip and a spring (2). A silicone rubber o-ring is placed in the circular groove (3) around the cantilever to provide an enclosed fluid environment between the fluid cell and the scanner. Two channels (4) make the exchange of the enclosed liquid possible. The piezoelectric material used to excite the cantilever is located above one of supporting holes (5) and its wires pass through the fluid cell to the connecting chip (6). In this way, the whole electronic system is completely insulated from the fluid.

**Figure 3.**
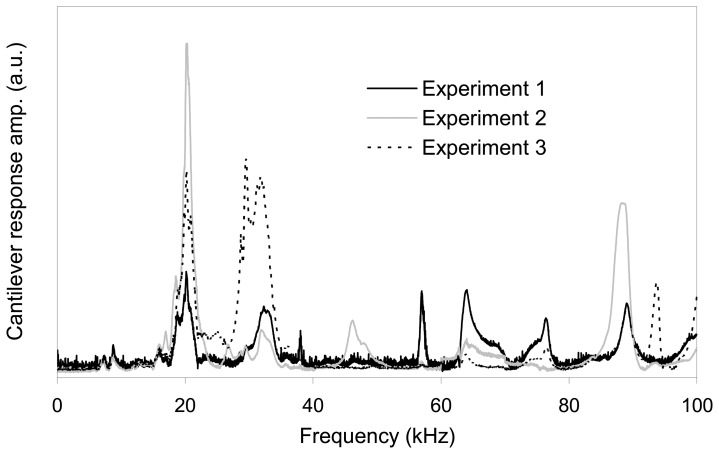
Frequency responses of cantilever 1 with different cantilever base and clip positions (in water). The fundamental resonant frequency of this cantilever is approximately 20 kHz.

**Figure 4.**
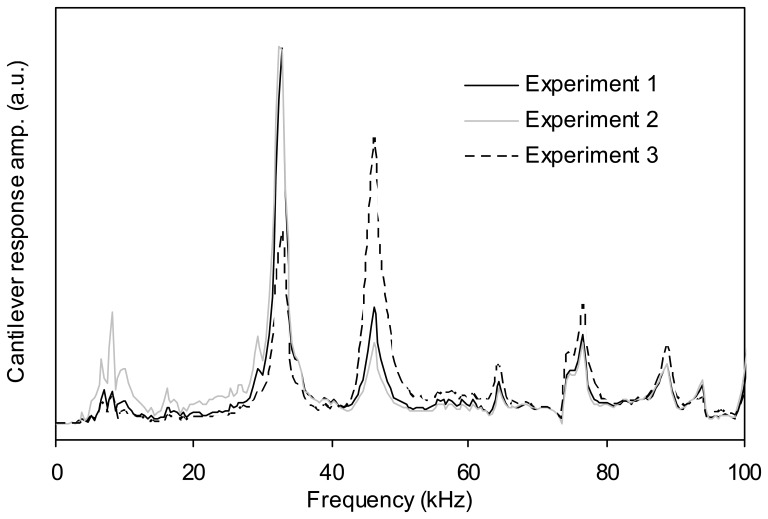
Repeatability of the frequency response of cantilever 2 when glued to the fluid cell (in water). The fundamental resonant frequency of this cantilever in water is approximately 35 kHz.

In our studies we have used four different cantilevers selected according to the requirements of each experiment. Cantilevers 1 and 2 were used in the initial experiments (results in Figures 3 and 4) to observe the effect of each modification to the fluid cell to the quality of the response. It was not possible to retain these cantilevers as they broke during the study and therefore their dimensions have not been recorded. Additionally, their fundamental resonant frequencies in water are different; approximately 20 and 35 kHz for cantilevers 1 and 2 respectively. For the quantitative studies (results presented in Figures 6, 7, 8 and 9) we used model CLFC-NOBO cantilever chips from Veeco. Each chip contains 3 cantilevers (short, medium and long) of equal and homogeneous width and material properties and slightly different thicknesses (Table 1). The exact thicknesses of each cantilever, which ranged between 1.8 and 2.2 μm, were determined from its resonance peak in air. Experiments were performed with the long cantilever (cantilever 3, length = 397 μm) and the medium cantilever (cantilever 4, length = 197 μm). Additionally, three water-glycerin solutions were used in the experimental studies (Table 2).

As is clear from Figure 2, the entire fluid cell is vibrated in order to excite the cantilever in the tuning process and the frequency sweep experiment. This is in contrast to the regular tip holders used in air or vacuum or other commercial fluid cells, in which the piezoelectric actuator is located directly under the cantilever base and causing only the cantilever to oscillate. This means that while the regular tip holders can achieve ideal acoustic excitation; this fluid cell cannot, as we will now demonstrate.

This fluid cell design has several drawbacks. One problem is the holding clip because first of all, its spring is not strong enough to secure the cantilever base tightly, and secondly it does not necessarily hold the cantilever such that its axis is perpendicular to the clip rod. Since the surface of the cantilever chip is sloped, any configuration other than perpendicular results in only a single point of contact reducing the overall stability of the connection. The other end of the clip, which is above the fluid cell, can easily be moved or rotated during handling and mounting of the fluid cell on the AFM head thus changing the connection between the clip and the cantilever base. Moreover this can result in displacement of the cantilever chip in its groove and consequent misalignment of the laser beam from the AFM head. This is especially important because when the cantilever base moves to another position in its groove it creates a new vibrational system with a different frequency response. Therefore, the clip and spring system does not allow for reproducible experiments as shown in Figure 3. For these experiments we used cantilever 1, which has a fundamental resonant frequency of approximately 20 KHz in water. The fundamental resonant frequency in the three responses is constant at about 20 kHz. The position of this peak is unaffected by the cantilever chip location but its amplitude is significantly affected. We note that when attempting to study the rheological properties of fluids, both the shape and the location of the primary peak are important. Also, for the other system resonances in Figure 3, neither the position nor the amplitude of the peaks is constant and they strongly depend on the position of the clip and cantilever.

This problem was solved by removing the clip and gluing the cantilever base to the fluid cell using silicone glue [24]. As a result of this modification, some redundant peaks associated with the clip and spring were eliminated from the frequency response of the system and the reproducibility of the experiments was improved. It should be mentioned that the problem of irreproducibility is not completely solved because the positioning of the fluid cell in the AFM head and also the force applied by the grip over the cell cannot be exactly repeated by hand. However, these are relatively minor effects and by gluing the cantilever base to the fluid cell, we can improve the repeatability of the frequency response considerably. Figure 4 demonstrates the improvement in repeatability in the frequency response of a cantilever when glued to the fluid cell. For these experiments a new cantilever was used, namely cantilever 2 which had a fundamental resonant frequency in water of about 35 kHz.

**Figure 5.**
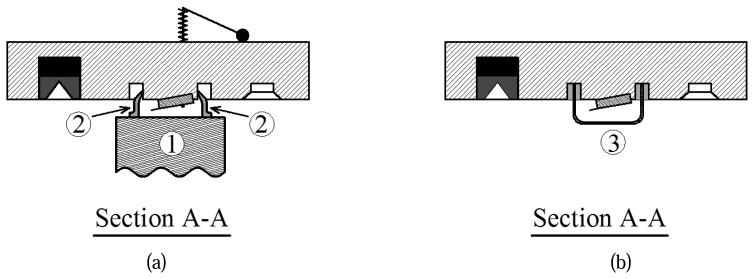
Cross section of the fluid cell defined in Figure 2 (a) before modification and (b) after modification. In this picture, (1) is the scanner, (2) is the o-ring, and (3) is the reservoir.

**Figure 6.**
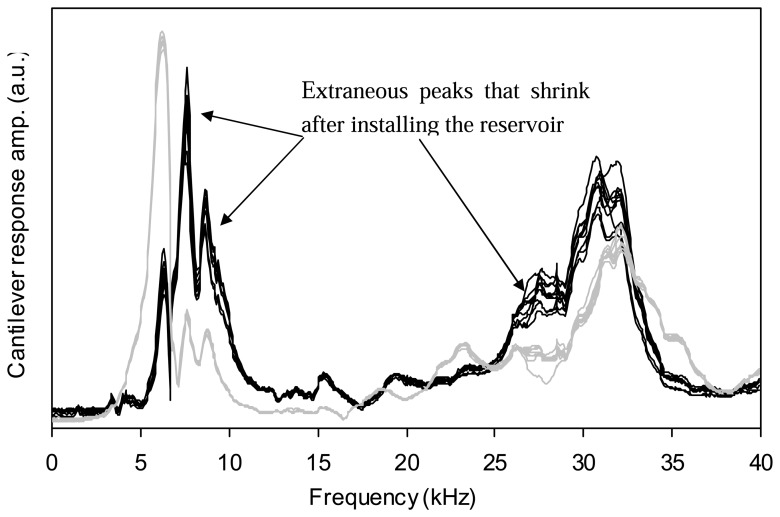
Frequency responses of cantilever 3 in 50% glycerin-water solution before (black line) and after (gray line) installing the reservoir. The cantilever is glued to the fluid cell.

The second problem arising from the design of the fluid cell is that it causes an unsteady, free surface flow of the fluid trapped between the cell and scanner (See Figure 5a). As mentioned previously, the piezoelectric actuator excites the cantilever through the movement of its base via vibration of the entire fluid cell. The large moving surface of the fluid cell also generates an unsteady flow in the fluid which affects the vibration of the cantilever and is in fact another source of excitation for the cantilever. This means that the cantilever is excited not only by the movement of its base (structure-borne excitation), but also by the unsteady fluid motion (fluid-borne excitation) resulting in additional resonance peaks in the frequency response (see Figure 6). The same problem was encountered by Xu and Raman [21], who used another type of commercial fluid cell from Agilent.

This problem can be solved by making a small fluid reservoir from glass and gluing it into o-ring groove of the fluid cell as shown in Figure 5b. The reservoir can be filled and emptied using the inlet and outlet channels of the fluid cell. If the reservoir is filled completely with liquid, then the fluid inside the reservoir has almost the same velocity as the fluid cell. In other words, the relative motion of the fluid due to excitation of the fluid cell is very small and does not affect the vibration of the cantilever. Many of the spurious peaks in the frequency response of the cantilever then shrink. Figure 6 shows the frequency responses of a cantilever in a 50% glycerin-water solution with and without the reservoir attached to the fluid cell. It should be emphasized that the fluid cell must be completely filled and free of bubbles and in order to accomplish this, the fluid must be degassed before filling the reservoir. When the reservoir is attached, the tip holder can only be used for rheological measurements as imaging would not be possible. For these experiments cantilever 3 was used. The installation of the reservoir causes shrinkage in the redundant peaks at 7, 9 and 27 kHz frequencies as shown by arrows in Figure 6.

The last and most important problem with the fluid cell design is that the measured vibration response is the combination of the cantilever vibration and the fluid cell vibration. The response of the fluid cell itself to the excitation is frequency dependent and not the same as the movement of the piezoelectric actuator. This means that the driving motion experienced by the cantilever is not the ideal constant amplitude sine wave. Therefore the presence of the fluid cell and anything else between the piezoelement and the cantilever base make it impossible to measure the real frequency response of the cantilever.

In order to experimentally verify the above theory, we measured the response of cantilevers 3 and 4 in three different solutions of glycerin and water using the modified fluid cell. Also after filling the reservoir, the inlet and outlet channels were blocked to prevent any evaporation. In this way we can be sure that fluid borne excitation of the cantilever is negligible. Recall that the properties of the cantilever and the surrounding liquids are summarized in Tables 1 and 2.

**Figure 7.**
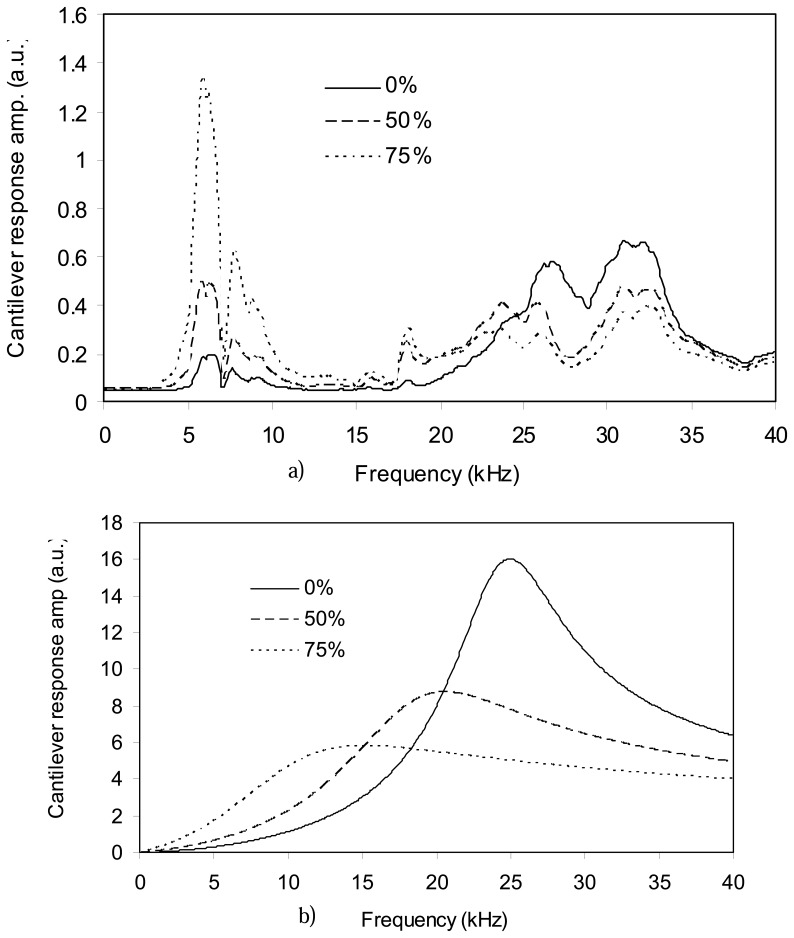
Response of cantilever 4 in three solutions of glycerin and water; a) measured by AFM optics, b) determined theoretically.

Figure 7a shows the responses of cantilever 4 observed by the AFM optics. The drive amplitude in all the experiments was the same and constant. Although the shapes of these responses are different, the positions of their peaks are at the same frequency. On the other hand, Figure 7b shows the theoretical responses of such a cantilever in the liquids based on the equations 8 and 9. For the theoretical response, the cantilever base was forced with a displacement amplitude of one at all frequencies, *Y(ω)*=*1*.

**Figure 8.**
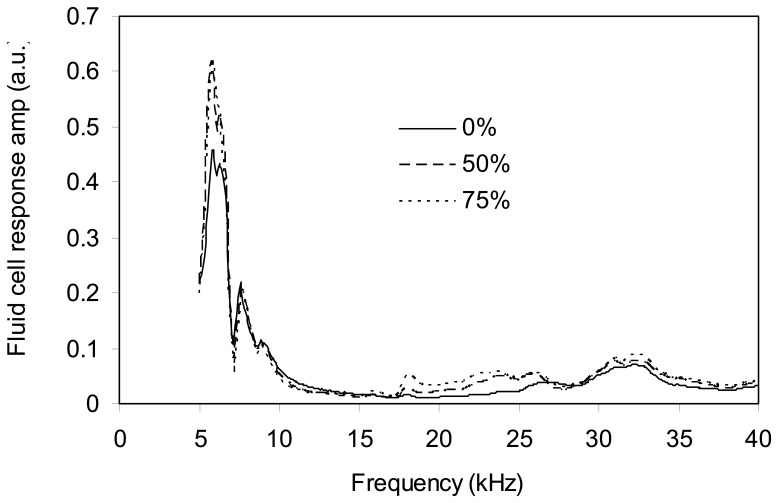
Fluid cell frequency responses when containing solutions of glycerin and water, observed with cantilever 4.

**Figure 9.**
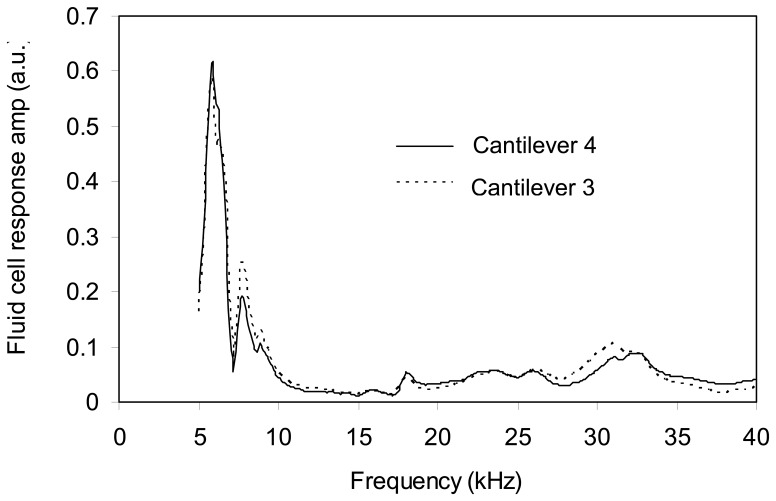
Fluid cell frequency responses obtained from the excitation of two different cantilevers in 75% glycerin-water solution. Cantilevers 3 and 4 have lengths of 397 and 197 mm respectively.

Comparing Figures 7a and 7b, one can find no similarity between the theoretical and experimental responses of the cantilever. However, when the experimental response in each liquid is divided by its ideal acoustic response, the results are the same for all liquids. These results, shown on Figure 8, are the response of the fluid cell at the cantilever base to the excitation from the piezoelement. The three curves are the same because the vibrational characteristics of the fluid cell are mainly dependant upon the elasticity and mass of the fluid cell. And in our case, the type of fluid affects only the mass of the fluid cell. However, since the densities of the fluids studied here are very close and since the volume of the reservoir is small, the total mass variation is negligible and therefore, the vibrational characteristics of the fluid cell are independent of the fluid it contains.

Based on the results shown on Figures 7 and 8, the experimental response to a specific excitation, *I(ω)*, in the absence of fluid born excitation, can be written in the form:

(11) $$W_{\text{exp}}\;(x,\omega )=T_{C}\;(x,\omega )\times T_{F}\;\left(\omega \right)\times I\;\left(\omega \right)$$

where *T_F_(ω)* is defined as an experimentally obtained function that transfers *I(ω)* to the frequency response of the fluid cell itself, *T_C_(x,w)* is the transfer function for the ideal damped response of the cantilever (Equation 9) and together their product represents the experimental response of the cantilever *W_exp_(x,ω)*. Note that the cantilever is excited by the function *Y(w)*=*T_F_(ω)*×*I(ω)* illustrating that ideal acoustic excitation can only be achieved if the fluid cell is designed such that *T_F_(ω)* is constant.

To further verify the linearity of the model in Equation 11, experiments were conducted using the longer cantilever 3 and the same fluid cell with a 75% glycerin-water solution. Results for these experiments show an identical fluid cell frequency response.

Equation 11 can also be used to understand that the liquid damped dynamics of the cantilever, *T_C_(x,w)*, act to amplify the dynamics of the fluid cell. When the cell is filled with air or another gas the damped cantilever response contains only sharp resonance peaks at higher frequencies and thus acts to filter out the dynamics of the cell itself.

The results presented here prove that with this type of fluid cell the frequency response is dominated by the dynamics of the cell itself rather than the cantilever and that fluid borne excitation is less important than previously thought. This problem can only be solved by placing the peizoelectric actuator directly under the cantilever base as in the regular tip holders. Maali *et al.* [25] modified a regular commercial cantilever holder to improve the acoustic excitation of cantilevers in liquids. They insulated the piezoelectric element by a thin film of Teflon and also installed a small piece of microscope glass to cover the liquid just above the cantilever and the piezoelectric element. Their experimental results follow the theoretical predictions for ideal acoustic excitation very well indicating that such experiments are feasible. This indicates that commercial suppliers of fluid cells must rethink their designs with the dynamic characteristics in mind.

We have further demonstrated that the important design issues for fluid cells are: (1) the method of attaching the cantilever chip to the cell, (2) the location of the piezoelectric actuator and (3) the occurrence of fluid borne excitation. Of these issues, the first relates to repeatability and the second and third determine the potential for achieving ideal acoustic actuation. If the actuator is placed exactly at the base of the cantilever following the approach of Maali *et al.* [25], one can design a system where only the cantilever is excited and fluid borne excitation is not occurring. In that case only the first two design issues mentioned above are relevant.

## 4. Conclusions

The frequency response of AFM microcantilevers, in a commercial fluid cell, was investigated while the cantilevers were immersed in different liquids. The dynamic characteristics of the fluid cell were determined by combining the experimental and theoretical results. It was shown that in fluid cells in which the piezoelectric element is removed from the cantilever base, ideal acoustic excitation cannot be achieved. Moreover in this case, the measured frequency response is dominated by the dynamics of the fluid cell potentially leading to significant misinterpretation of data. Contrary to previous reports, fluid borne excitation is shown to be a less significant effect.

## Figures and Tables

**Table 1.** Cantilever properties.

**Description**	**Value**
**Width**	29 (mm)
**Thickness**	2 (mm)
**Mass density**	2300 (kg/m^3^)
**Young's modulus**	170 (GPa)

**Table 2.** Properties of glycerin-water solutions.

**Glycerin content(wt%)**	**Density (kg/m^3^)**	**Viscosity (mPa.s)**
0%	997	0.8628
50%	1122	4.747
75%	1191	25.49
